# Supplementary material for: Proteolysis-targeting chimera against BCL-XL destroys tumor-infiltrating regulatory T cells
Source: Nat Commun. 2021 Feb 24;12:1281. doi: 10.1038/s41467-021-21573-x (PMC7904819; doi:10.1038/s41467-021-21573-x)
Supplement: Supplementary file 1 — Supplementary Information File [file 41467_2021_21573_MOESM1_ESM.docx]

**Supplementary Information**

Proteolysis-targeting chimera against BCL-X_L_ destroys tumor-infiltrating regulatory T cells

Authors: Ryan Kolb^1,2,†^, Umasankar De^1,†^, Sajid Khan^3^, Yuewan Luo^1^, Myung-Chul Kim^1^, Haijun Yu^1^, Chaoyan Wu^1^, Jiao Mo^1^, Xin Zhang^3^, Peiyi Zhang^4^, Xuan Zhang^4^, Nicholas Borcherding^5^, Daniel Koppel^2,6^, Yang-Xin Fu^7^, Song Guo Zheng^8^, Dorina Avram^2,9,10^, Guangrong Zheng^2,4^, Daohong Zhou^2,3,*^, Weizhou Zhang^1,2,*^

^1^Department of Pathology, Immunology, and Laboratory Medicine, School of Medicine, University of Florida, Gainesville, FL 32610, USA

^2^University of Florida Health Cancer Center, University of Florida, Gainesville, FL 32610, USA

^3^Department of Pharmacodynamics and ^4^Department of Medicinal Chemistry, School of Pharmacy, University of Florida, Gainesville, FL 32610, USA

^5^Department of Pathology, University of Iowa, Iowa City, IA 52241

^6^Department of Chemistry, College of Liberal Art and Sciences, University of Florida, Gainesville, FL 32610, USA

^7^ Department of Pathology, University of Texas Southwestern Medical Center, Dallas, TX 75235-9072, USA

^8^Department of Internal Medicine, Ohio State University College of Medicine and Wexner Medical Center, Columbus, OH 43210

^9^Department of Anatomy and Cell Biology, University of Florida College of Medicine, 32610, Gainesville, FL 32610

^10^Department of Immunology, Moffitt Cancer Center, Tampa, FL 33612

^†^These authors contributed equally: Ryan Kolb and Umasankar De

^*^These authors jointly supervised this work: Daohong Zhou and Weizhou Zhang.

Correspondence: W.Z.: [zhangw@ufl.edu](mailto:zhangw@ufl.edu); D.Z.: [zhoudaohong@cop.ufl.edu](mailto:zhoudaohong@cop.ufl.edu)

Running title: Targeting tumor-infiltrating Tregs for cancer therapy

**Supplementary Table 1. Statistics for the expression of BCL-2 family genes in TI-Tregs and splenic Tregs from MC38 tumor model.**

| Gene | Significant? | P value | Mean of Spleen | Mean of Tumor | Difference | SE of difference |
| --- | --- | --- | --- | --- | --- | --- |
| *Bcl2* | Yes | <0.000001 | 6.172 | 3.742 | 2.43 | 0.1782 |
| *Bcl2l1* | Yes | <0.000001 | 4.441 | 6.718 | -2.277 | 0.07499 |
| *Bcl2l2* | Yes | 0.000178 | 2.056 | 3.211 | -1.155 | 0.1892 |
| *Mcl1* | Yes | 0.000003 | 7.734 | 8.843 | -1.109 | 0.1085 |
| *Bcl2a1a* | Yes | <0.000001 | 1.401 | 5.977 | -4.576 | 0.2687 |
| *Bcl2l12* | No | 0.477723 | 2.771 | 2.869 | -0.09895 | 0.1336 |
| *Bax* | Yes | <0.000001 | 5.279 | 6.102 | -0.8227 | 0.05227 |
| *Bak1* | Yes | <0.000001 | 4.191 | 5.573 | -1.382 | 0.07846 |
| *Bok* | Yes | 0.000139 | 0.7015 | 3.021 | -2.319 | 0.3675 |
| *Bcl2l13* | No | 0.086341 | 3.112 | 3.198 | -0.08592 | 0.04463 |
| *Bcl2l14* | Yes | 0.008823 | 0.9233 | 1.988 | -1.065 | 0.3199 |
| *Bcl2l15* | Yes | 0.002211 | 0.725 | 0.2764 | 0.4486 | 0.1061 |
| *Bnip2* | Yes | <0.000001 | 5.225 | 6.258 | -1.033 | 0.06708 |
| *Bad* | Yes | <0.000001 | 3.207 | 4.386 | -1.179 | 0.07819 |
| *Bbc3* | Yes | 0.000521 | 4.389 | 3.802 | 0.5872 | 0.1116 |
| *Bcl2l11* | No | 0.23571 | 5.638 | 5.818 | -0.1806 | 0.1421 |
| *Bid* | No | 0.085585 | 3.93 | 3.776 | 0.1543 | 0.07993 |
| *Bmf* | Yes | 0.009867 | 1.825 | 2.798 | -0.9735 | 0.2988 |
| *Bik* | No | 0.780905 | 0.9944 | 0.946 | 0.04837 | 0.1688 |
| *Bnip1* | No | 0.053678 | 3.585 | 3.836 | -0.2505 | 0.1129 |
| *Bnip3* | Yes | <0.000001 | 2.052 | 7.795 | -5.743 | 0.2081 |
| *Bop1* | No | 0.507229 | 4.725 | 4.671 | 0.05408 | 0.0783 |
| *Pmaip1* | Yes | <0.000001 | 3.538 | 5.135 | -1.597 | 0.1353 |

Related to Figure 1b.


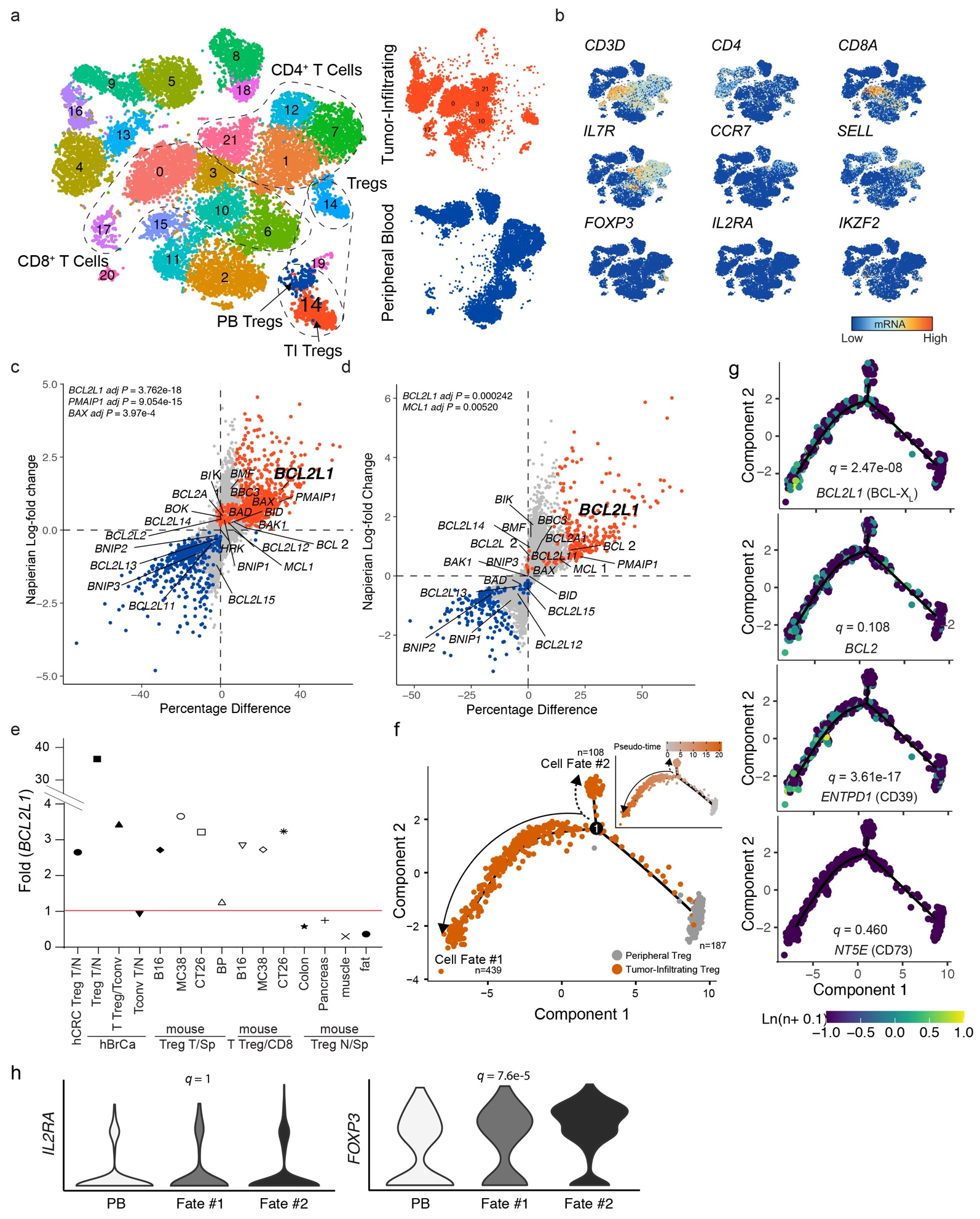


**Supplementary Figure 1. BCL-X_L_ levels are higher in tumor-infiltrating (TI) Tregs than Tregs from peripheral blood (PB).** a. tSNE plot of immune cells from 3 ccRCC tumors with immune cells from paired peripheral blood (PB) of the same patients (GSE121638: <https://www.ncbi.nlm.nih.gov/geo/query/acc.cgi?acc=GSE121638>). The dotted black line separates major T cell subtypes. A total 22 clusters are depicted and the Treg cluster 14 includes both TI- and PB-Tregs. b. tSNE projection with highlighted expression of T cell markers used to define sub-populations of T cells. c-d. Graphs depicting the log-fold change and percent difference in the number of cells that express each gene in TI-Tregs *versus* PB-Tregs, as determined from scRNAseq of TI and PB lymphocytes from RCC (c) and HCC (GSE98638: https://www.ncbi.nlm.nih.gov/geo/query/acc.cgi?acc=GSE98638) (d) patients. Members of the *BCL2* gene family are indicated, with *BCL2L1* highlighted. Colored points are indicating genes that are significantly (Bonferroni-adjusted p-value < 0.05) upregulated (red) or downregulated (blue) in TI-Tregs. e. Linear fold difference in *BCL2L1* expression in Tregs from various species, tumor types, and normal tissues, relative to the indicated cell populations or the counterpart cell types from the spleen from published GEO dataset (GSE116347: <https://www.ncbi.nlm.nih.gov/geo/query/acc.cgi?acc=GSE116347>). h, human; m, mouse; CRC, colorectal cancer; BrCa, breast cancer; T, tumor; Sp, spleen; N, normal tissues. All the mean fold differences are for pairs of tumor and normal tissue, pairs of tumor and spleen, or pairs of normal and spleen. Red line: fold = 1. f. Trajectory manifold of Tregs from the ccRCC using the Monocle 2 algorithm, solid and dotted lines represent distinct cell trajectories/fates defined by expression profiles. Number of Tregs within each cell fate is labelled (GSE121638). g. Cell trajectory projections of transcriptional changes for the indicated genes based on the manifold (GSE121638). h. Expression levels of *IL2RA* (CD25) and *FOXP3* at different Treg populations (GSE121638). PB: peripheral blood. (g-h). Significance is based on one-way ANOVA test between the 3 branches of Tregs with adjusted *P* values (*q*) indicated.


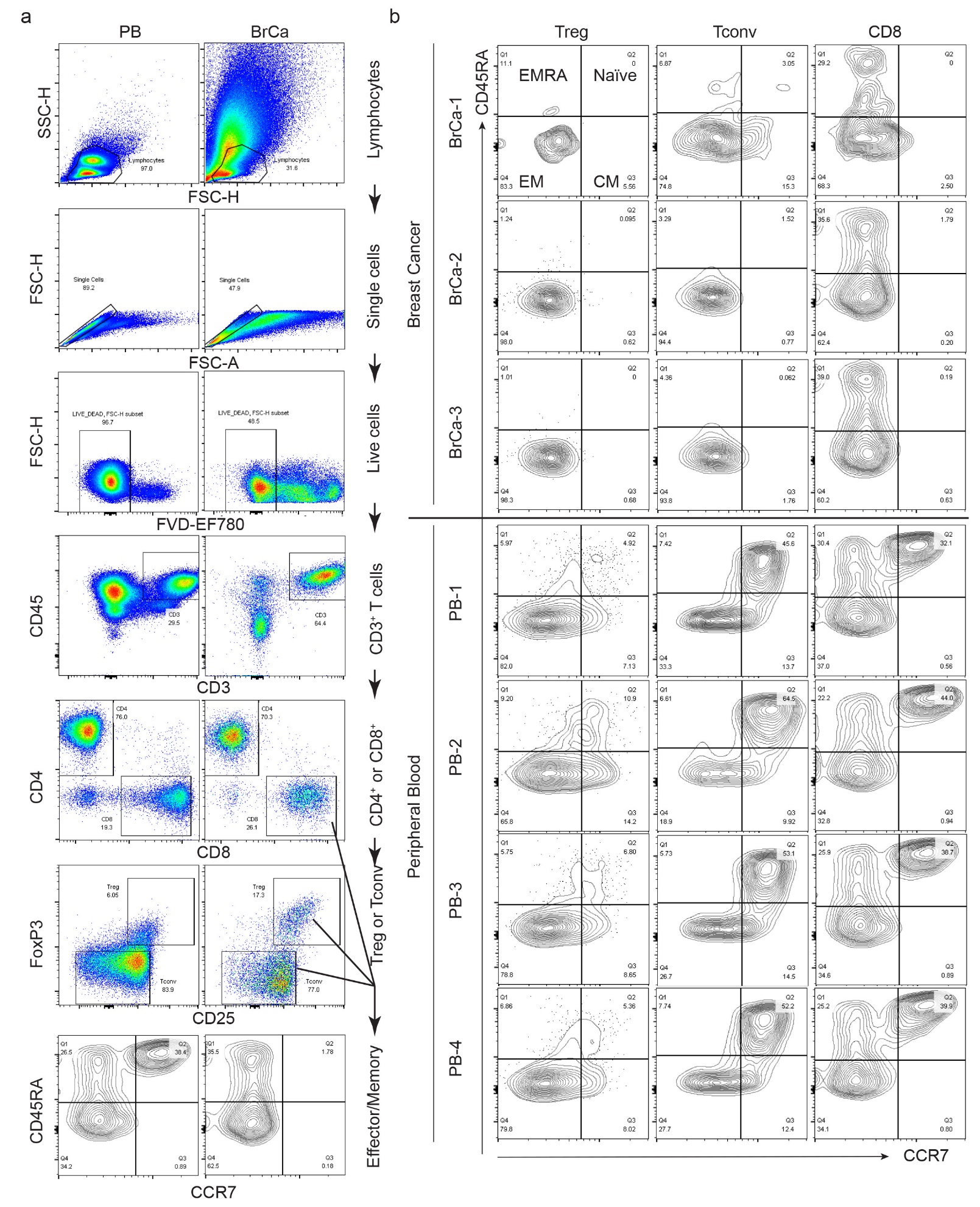


**Supplementary Figure 2. Gating scheme of flow cytometry in the human cancers.**

a. Gating scheme of flow cytometry for the identification of T cell populations in human cancers used in Fig. 1c-f, Fig. 2a-d, Fig. 4h-i and Supplementary Fig. 2b; 3a-b,d,h; 4b-d. b. Effector/memory phenotypes of T cell populations from 3 human breast cancer specimens and 4 human peripheral bloods based on CD45RA and CCR7 expression. FVD-EF780, fixable viability dye eFluor 780. PB, peripheral blood; BrCa, breast cancer; EMRA, effector; EM, effector memory; CM, central memory.


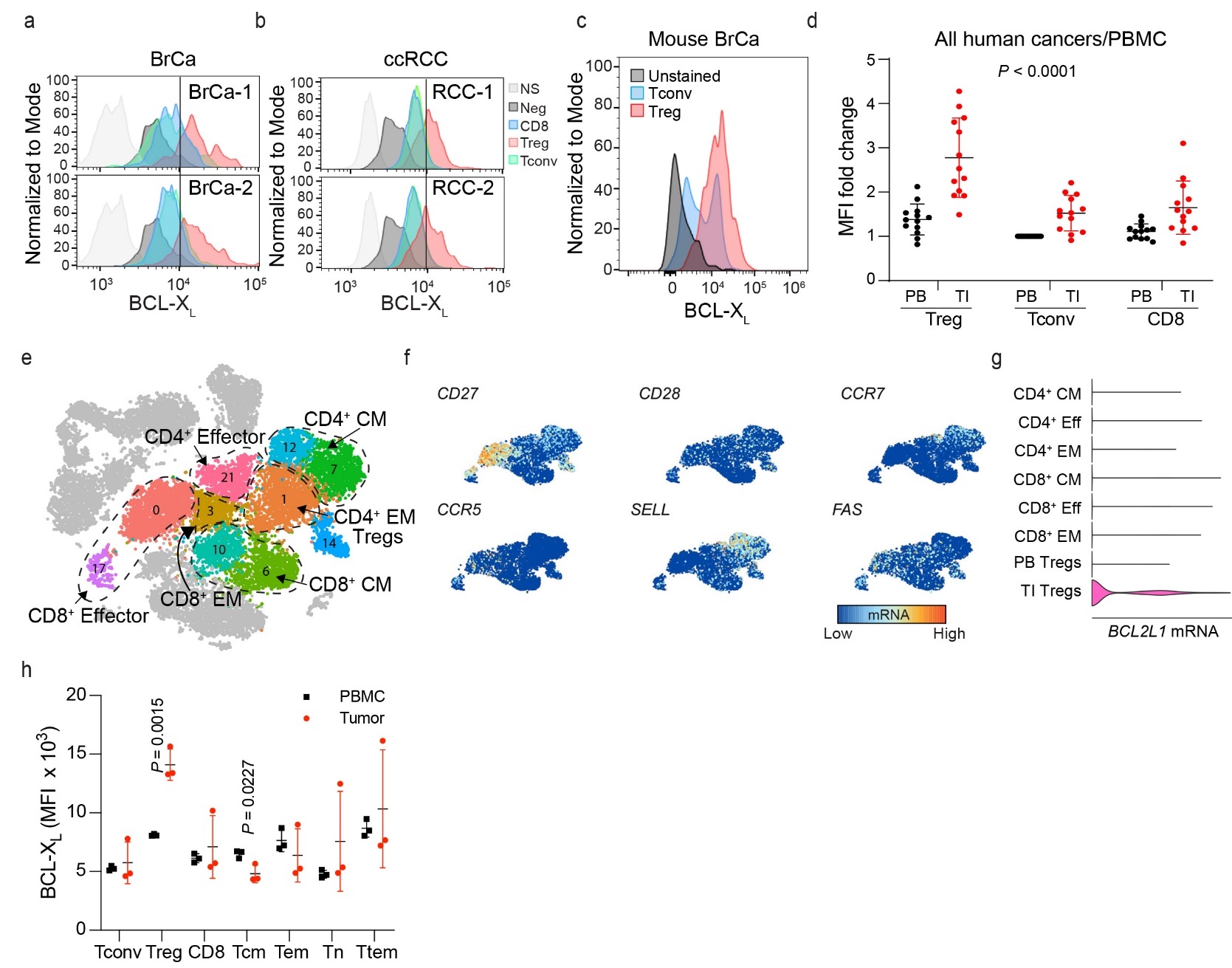


**Supplementary Figure 3. BCL-X_L_ is upregulated in TI-Tregs.**

1. Two representative histograms for BCL-X_L_ staining in human breast cancer (BrCa). b. Two representative histograms for BCL-X_L_ staining, depicting the levels of BCL-X_L_ staining of the indicated TI-T cells in human RCC patients. a-b. NS, non-staining; Neg, negative control, treated with DT-2216 to deplete BCL-X_L_ as negative control. c. Representative histogram depicting the level of BCL-X_L_ staining in Treg and Tconv cells isolated from mouse mammary tumors derived from Py8119 cells. d. Fold change of Median fluorescence intensity (MFI) of BCL-X_L_ staining ± s.d. in PB- or TI-Tregs, CD8^+^ and Tconv in these cancers (n=13 biological samples, including 9 paired BrCa and PBs; 2 paired ccRCC and PBs, 2 paired colon cancer and PBs). MFI is normalized to the MFI of PB-Tconv of the individual pair. Two-sided one-way ANOVA test was performed. e-g. tSNE projection of T cell subtypes from ccRCC SCRC data (e) with highlighted expression of T cell effector and memory markers used to define sub-populations of T cells designated as CM for central memory, EM for effector memory or as effectors cells. (f). Violin plot showing *BCL2L1* expression within these different clusters of T cells based on effector/memory designation (g, *P* = 0.001, two-sided one-way ANOVA). h. Average MFI of BCL-X_L_ staining ± s.d. in the indicated PB- or TI-T cells from 3 paired human breast cancers and PB samples. Two-sided unpaired T test was performed.


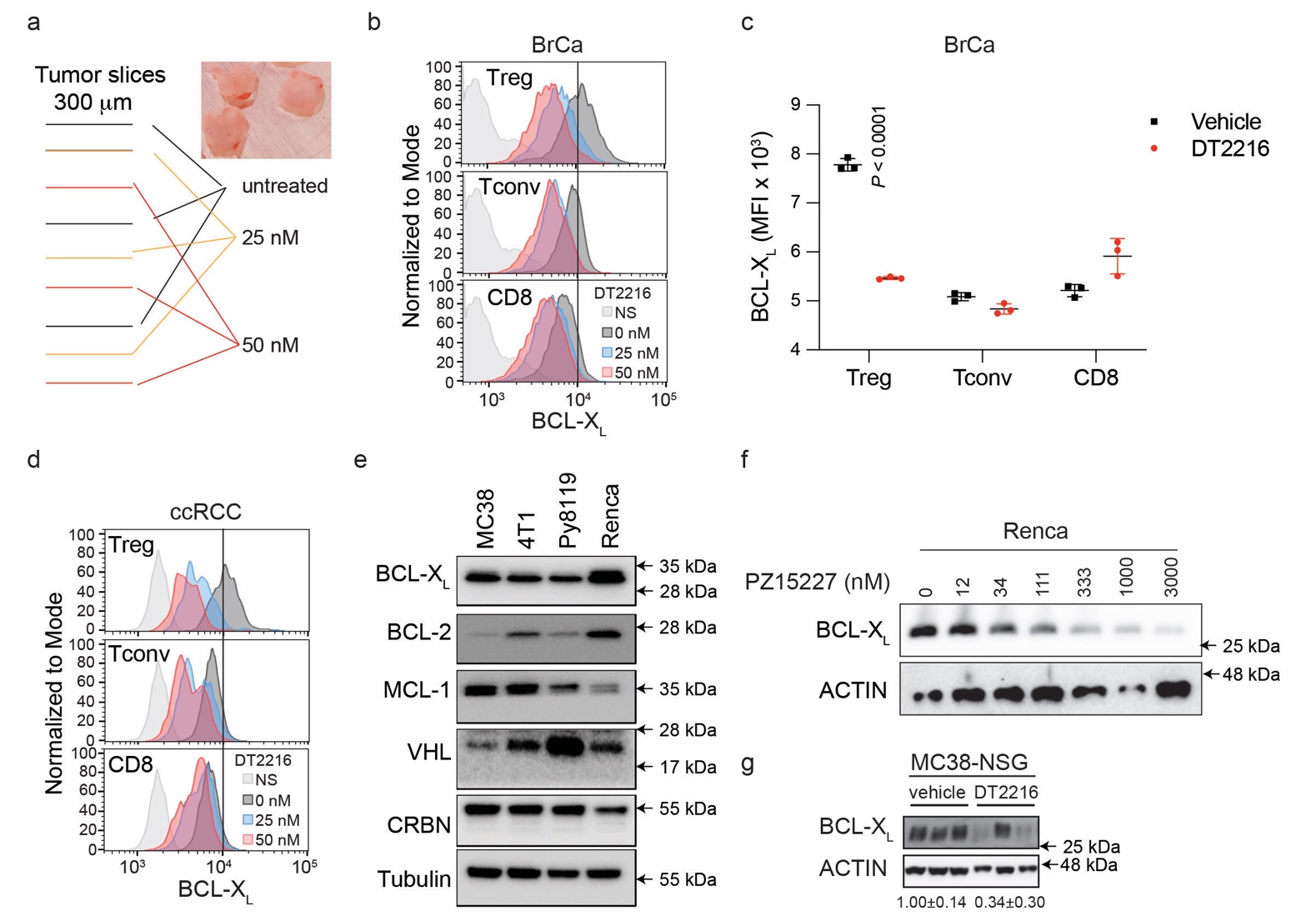


**Supplementary Figure 4. BCL-X_L_ PROTACs efficiently leads to the degradation of BCL-X_L_.**

a. Scheme for allocating tumor slices to test groups for culture and drug treatment strategies such as that used in Fig. 2; alternating 300 µm thick slices of human or mouse tumors are treated *ex vivo* with the indicated doses of DT2216. b. BCL-X_L_ levels in TI-Tregs, Tconv or CD8^+^ T cells in *ex vivo* slice cultures of human breast cancer, following treatment with 50 nM of DT2216 for 48 hrs, related to Fig. 2a-b. c. BCL-X_L_ levels in TI-T cells in *ex vivo* slice cultures of human breast cancer specimens, showing MFI of BCL-X_L_ in the indicated TI-T cells ± s.d. (n=3) in a separate specimen, following treatment with DT2216 for 48 hrs. Two-way unpaired T test was used. d. BCL-X_L_ levels in TI-Tregs, Tconv or CD8^+^ T cells in *ex vivo* slice cultures of human renal cancer, following treatment with DT2216 for 48 hrs, related to Fig. 2c-d. e. Immunoblot of BCL-2 family proteins (BCL-X_L_, BCL-2 and MCL-1) and E3 ubiquitin ligases (VHL and CRBN) in four mouse tumor cell lines. Tubulin serves as loading control. n=2 biological replicates. f. Immunoblot of BCL-X_L_ in the indicated mouse cancer cells following 16-hour treatment with the indicated concentration of the PROTAC PZ15227. ACTIN serves as the loading control. n=2 biological replicates. g. Immunoblot of BCL-X_L_ in MC38 tumors from NSG immunodeficient mice following weekly treatment with DT2216 at 7.5 mg/kg for 3 weeks. ACTIN serves as the loading control. n=3 biological replicates.


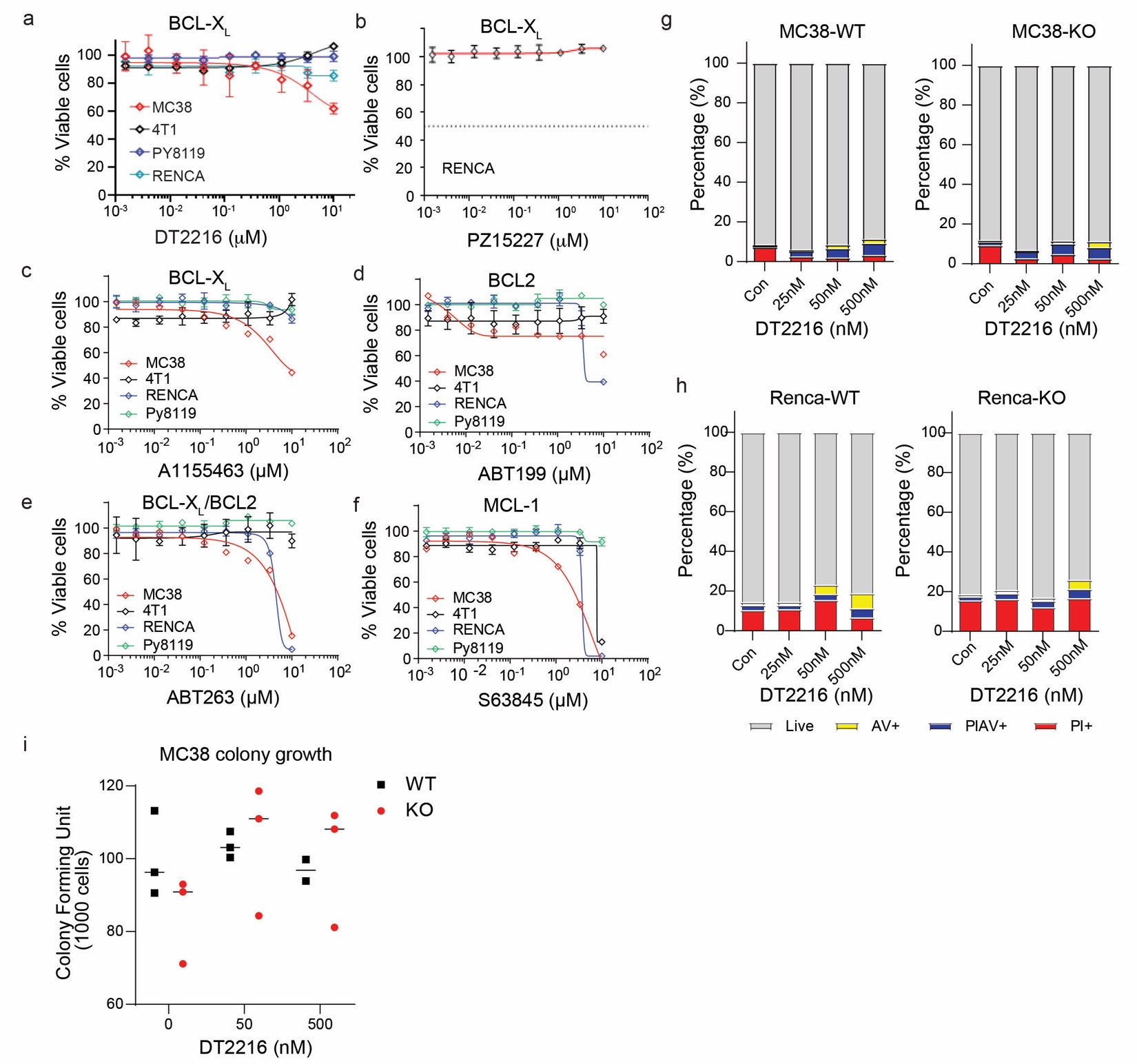


**Supplementary Figure 5. PROTAC-mediated BCL-X_L_-depletion does not impact cancer cell survival *in vitro*.**

a-f. Viability of indicated cells, as determined by MTS assay, following incubation with increasing concentrations of (a) DT2216, (b) PZ15227, (c) A1155463 (BCL-X_L_ selective), (d) ABT199 (BCL-2 selective), (e) ABT263 (BCL-X_L_ and BCL-2), or (f) S63845 (MCL-1 selective). The data are presented as mean ± s.d. (n = 5 biological replicates). g-h. Effects of BCL-X_L_ knockout (KO) on apoptosis *in vitro*, in MC38 and Renca cells, as assessed using Annexin V (AV) and propidium iodide (PI) staining (n=3 biological replicates). i. Effects of BCL-X_L_ KO on colony forming efficiency in soft agar (colony forming units calculated as per 1000 cells) (n = 3 biological replicates for all except n=2 for 500 nM-treated control group). All error bars represent standard deviation.


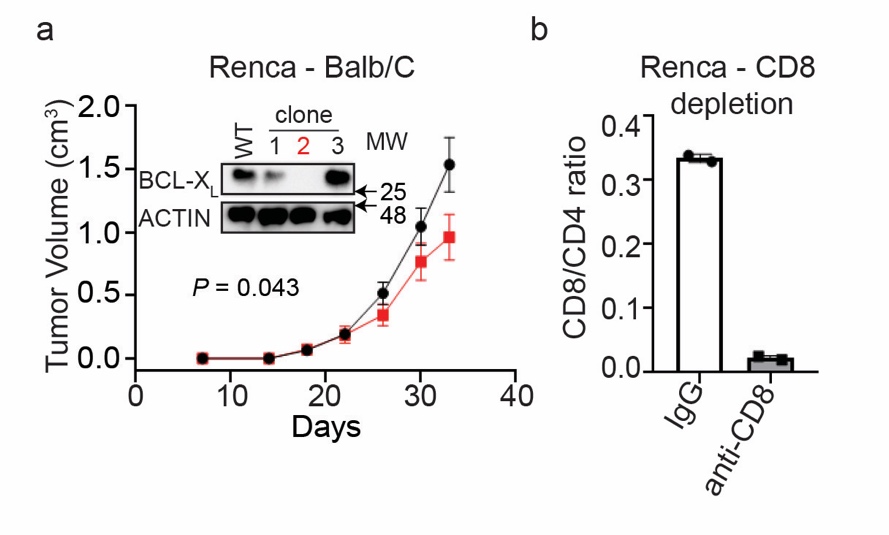


**Supplementary Figure 6. Depleting the tumor microenvironment of BCL-X_L_ reduces tumor growth in a CD8^+^ T cell dependent manner.** a. Effects of BCL-X_L_ knockout on tumor volume in Renca cell clones depicted in inset showing reduction in BCL-X_L_ levels. WT and Clone 2 cells were used for tumor growth, no treatment. Two-sided two-way ANOVA was performed and *P* value is indicated (n =7 biological replicates). Error bars represent standard error. b. Effectiveness of antibody-mediated CD8^+^ T cell depletion in Renca tumor-bearing mice, as assessed based on CD8/CD4 ratio. Error bars represent standard deviation (n=2 biological replicates of blood samples).


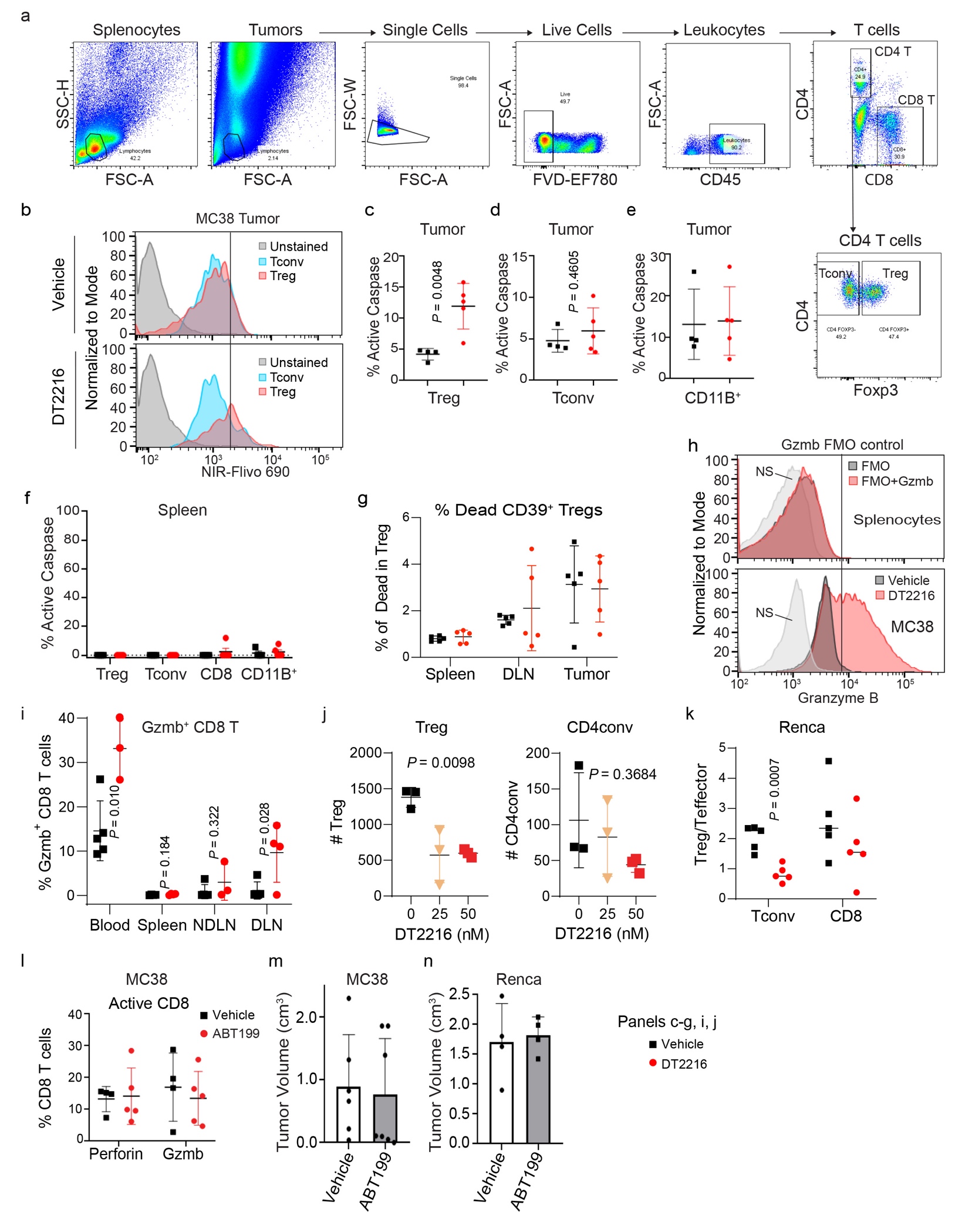


**Supplementary Figure 7. Treatment with the PROTAC DT2216 induces apoptosis of TI-Tregs.**

a. Gating scheme of flow cytometry for the identification of T cell subsets in MC38 tumors for Fig. 2e-f; Fig. 4; Supplementary Fig.3c; Fig.6b; Fig.7b-i; Fig.8-10. b. Representative histogram showing caspase activation (assessed using probe NIR-FLIVO 690) in TI-Tregs or TI-Tconv from MC38 tumors in mice 24 hours after treatment with PROTAC DT2216. c-e, Frequency of (c) Tregs, (d) Tconv and (e) CD11B^+^ myeloid cells with active caspase staining, expressed as percentage of NIR-FLIVO 690^+^ CD8^+^ T cells ± s.d (n=4 biological replicates for control and n=5 for DT2216-treated group). f. Percentage of cells within indicated splenocyte population in which caspases are active, expressed as percentage of NIR-FLIVO 690^+^ CD8^+^ T cells ± s.d. (n=5 biological replicates). g. Percentages of dead CD39^+^ TI-Tregs within total TI-Tregs, as indicated by positive staining of Fixable viability dye (FVD) eFluor-780, within MC38 tumors either treated with vehicle or DT2216 (mean ± s.d., n = 5 biological replicates). h. Related to Fig. 4f. Top panel: non-stained (NS), fluorescence-minus one (FMO) or FMO + granzyme B-BV421 on normal mouse splenocytes showing no active CD8 T cells. Bottom panel: representative histogram showing levels of granzyme B (Gzmb) in TI-CD8+ T cells from MC38 tumor-bearing mice treated with DT2216 (or vehicle). i. Number of granzyme B (Gzmb)^+^, CD8^+^ T cells (mean ± s.d.) within blood (n=5 in control and n=3 in DT2216 group, biological replicates), spleen (n=3 in control and n=3 in DT2216 group, biological replicates), non-draining lymph node (NDLN, n=3 in control and n=3 in DT2216 group, biological replicates) or DLN (n=3 in control and n=4 in DT2216 group, biological replicates) from MC38 tumor-bearing mice following treatment with DT2216 (or vehicle). j. Dose response to BCL-X_L_-targeted PROTAC DT2216 of (left) Tregs and (right) conventional CD4^+^ T cells (CD4conv) in slices of Py8119 tumors, as detected by flow cytometry of lymphocytes isolated from Py8119 tumor slices. Means ± s.d. are shown (n=3 biological replicates). k. Effects of DT2216 on the number of Tregs within Renca tumors, with TI-Treg/TI-Tconv or TI-Treg/TI-CD8 ratios shown (n=5 biological replicates). l. Effects of BCL-2 inhibitor ABT199 on percentages of Perforin^+^ and granzyme B (Gzmb)^+^ TI-CD8^+^ T cells among CD8^+^ T cells in MC38 tumors (mean ± s.d., n=4 biological replicates for control and n=5 for ABT199-treated group). m-n. Effects of BCL-2 inhibition on tumor volume in syngeneic (m) MC38 and (n) Renca mouse models. Shown are average MC38 tumor volume 45 days after injection, and average Renca tumor volume 38 days after injection, with inhibitor (ABT199) applied daily starting on day 17. Values are averages ± s.d. in C57BL/6 or Balb/C mice, respectively (n = 7 biological replicates). Error bars represent standard deviation. c-e, f-g, and i-n, two-sided unpaired t test was performed and *P* values are indicated.


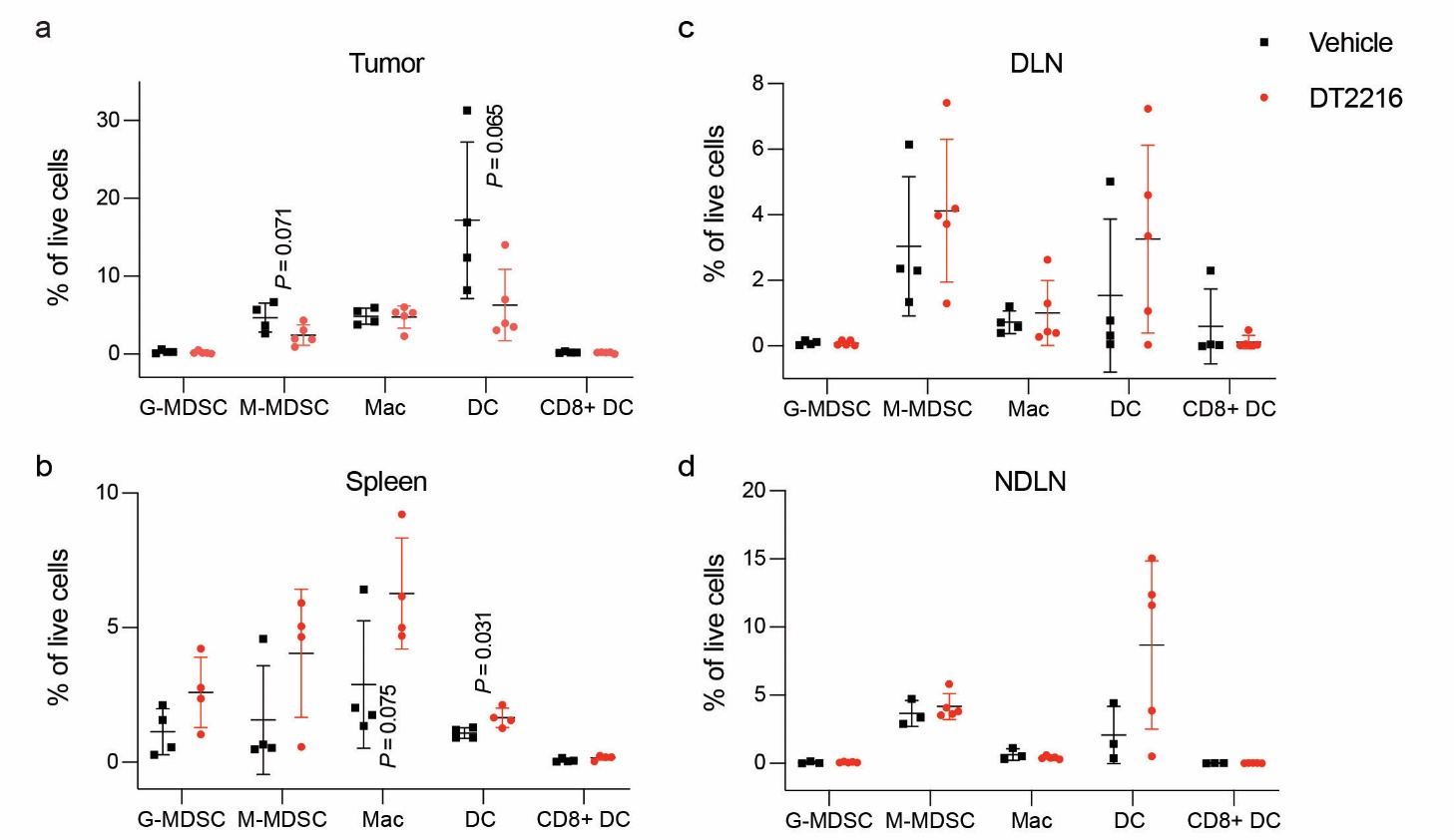


**Supplementary Figure 8. Treatment with the PROTAC DT2216 did not alter major myeloid cells in tumor-bearing mice.** a-d. Number of granulocytic or monocytic myeloid derived suppressor cells (G-MDSC, M-MDSC), macrophages, dendritic cells (DC) or CD8^+^ DC within (a) tumor (n=4 for control and n=5 for DT2216), (b) spleen (n=4 biological replicates), (c) DLN (n=4 for control and n=5 for DT2216) or (d) non-draining lymph node (NDLN, (n=3 for control and n=5 for DT2216)) from MC38 tumor-bearing mice following treatment with DT2216 (or vehicle). Error bars represent standard deviation. a-d, Two-sided unpaired t test was performed and *P* values are indicated.


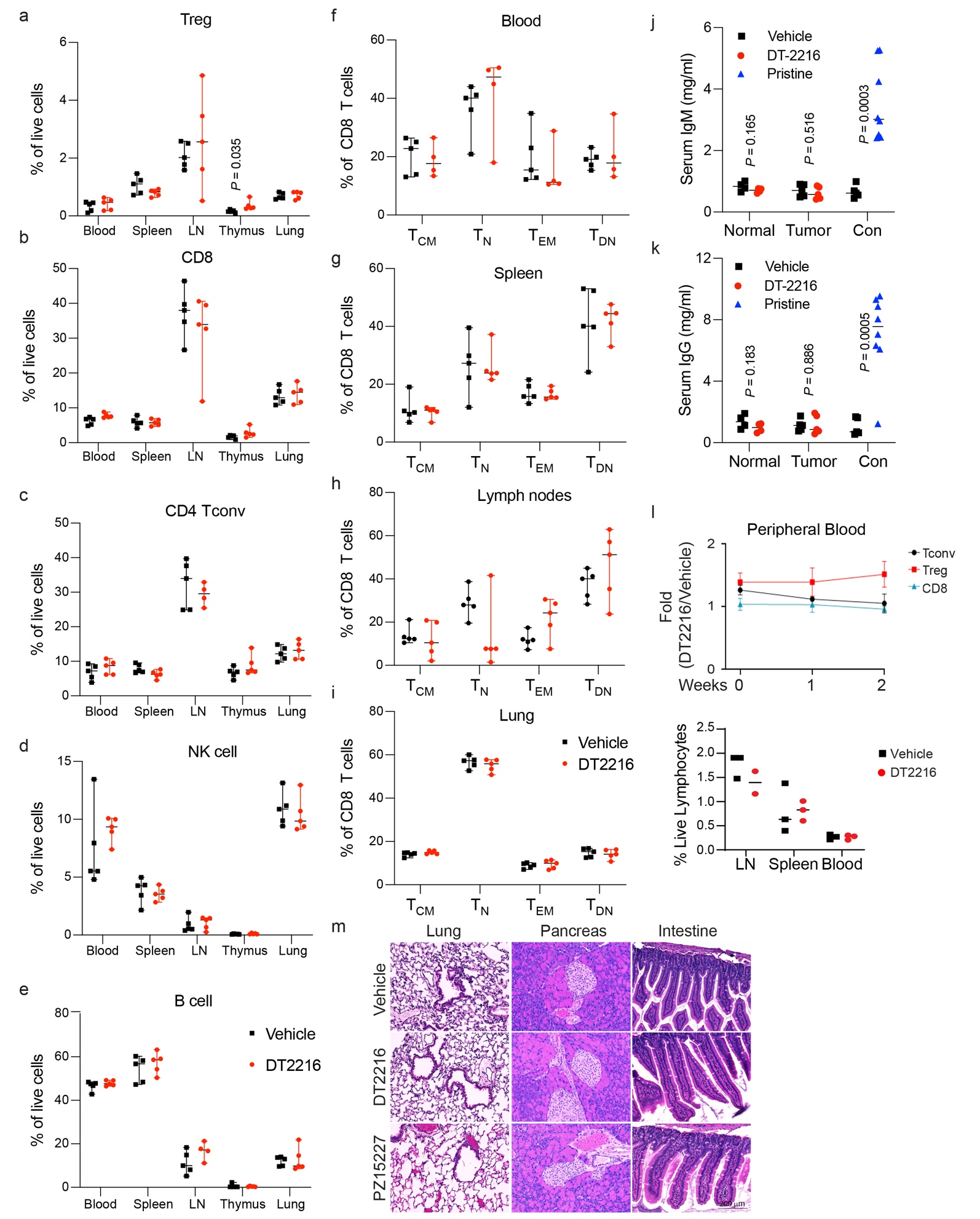


**Supplementary Figure 9. Treatment with the BCL-X_L_ PROTACs did not induce autoimmune phenotypes.** a-i. Effects of DT2216 on the number of different immune cells in normal female mice (n=5 biological replicates), as detected by flow cytometry of single cell suspensions from blood, spleen, lymph nodes, thymus and lung. j-k. Effects of DT2216 on total serum IgM (j) or IgG (k) in normal female and male mice (n=2 female and 3 male replicates), using non-treated age-matched mice as baseline control and pristane-treated mice as a positive control. l. (top) Effects of DT2216 on the number of PB-Tregs, PB-Tconv and PB-CD8^+^ T cells over time, and (bottom) effects of this treatment on the frequency of total Tregs from lymph node, spleen, and blood (assessed following third treatment). n=3 biological replicates for all groups except n=2 for LN group with DT2216 treatment. m. Representative hematoxylin and eosin staining of the indicated tissues from Renca tumor-bearing mice treated as indicated. a-i and j-k. Two-sided unpaired t test was performed and *P* values are indicated. All data points in this figure are mean ± s.d. with error bars representing standard deviation.


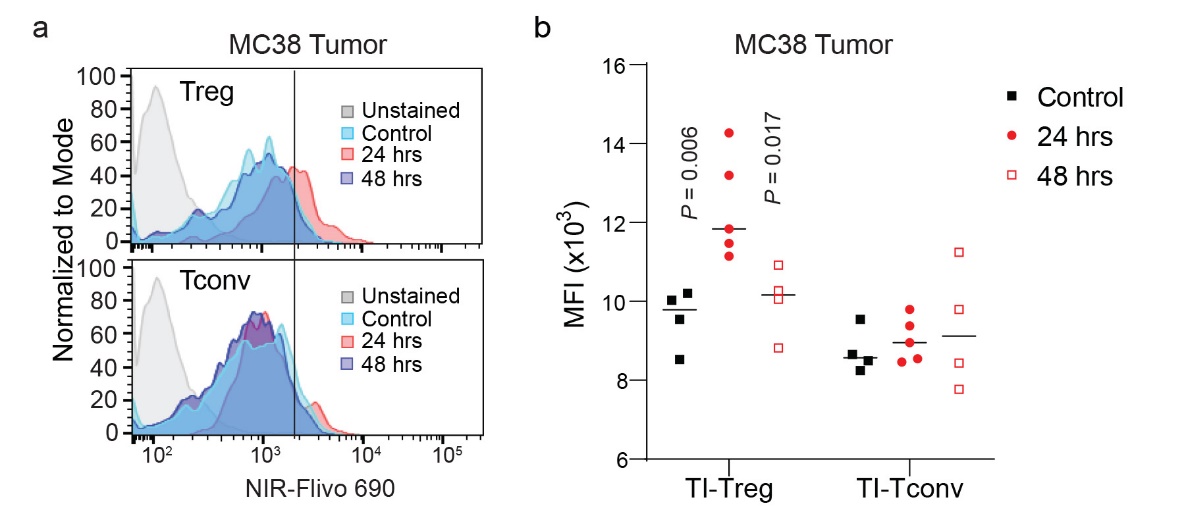


**Supplementary Figure 10. DT2216 induces transient apoptosis of TI-Tregs *in vivo*.** a. Representative histogram showing caspase activation (assessed using probe NIR-FLIVO 690) in TI-Tregs or TI-Tconv from MC38 tumors in mice 24 or 48 hours after treatment with PROTAC DT2216. Black line depicts MFI of TI-Tregs 24 hrs after DT2216 treatment. b. Amount of pan-caspase labeling in TI-Tregs or TI-Tconv from MC38 tumor-bearing mice following 24 or 48 hrs treatment with DT2216 (or vehicle) as assessed by flow cytometry. MFI of pan-caspase activation probe is shown (n=4 in control and 48 hrs groups; n=5 for 24 hrs group, all biological replicates). Two-sided unpaired t test was performed and *P* values are indicated.


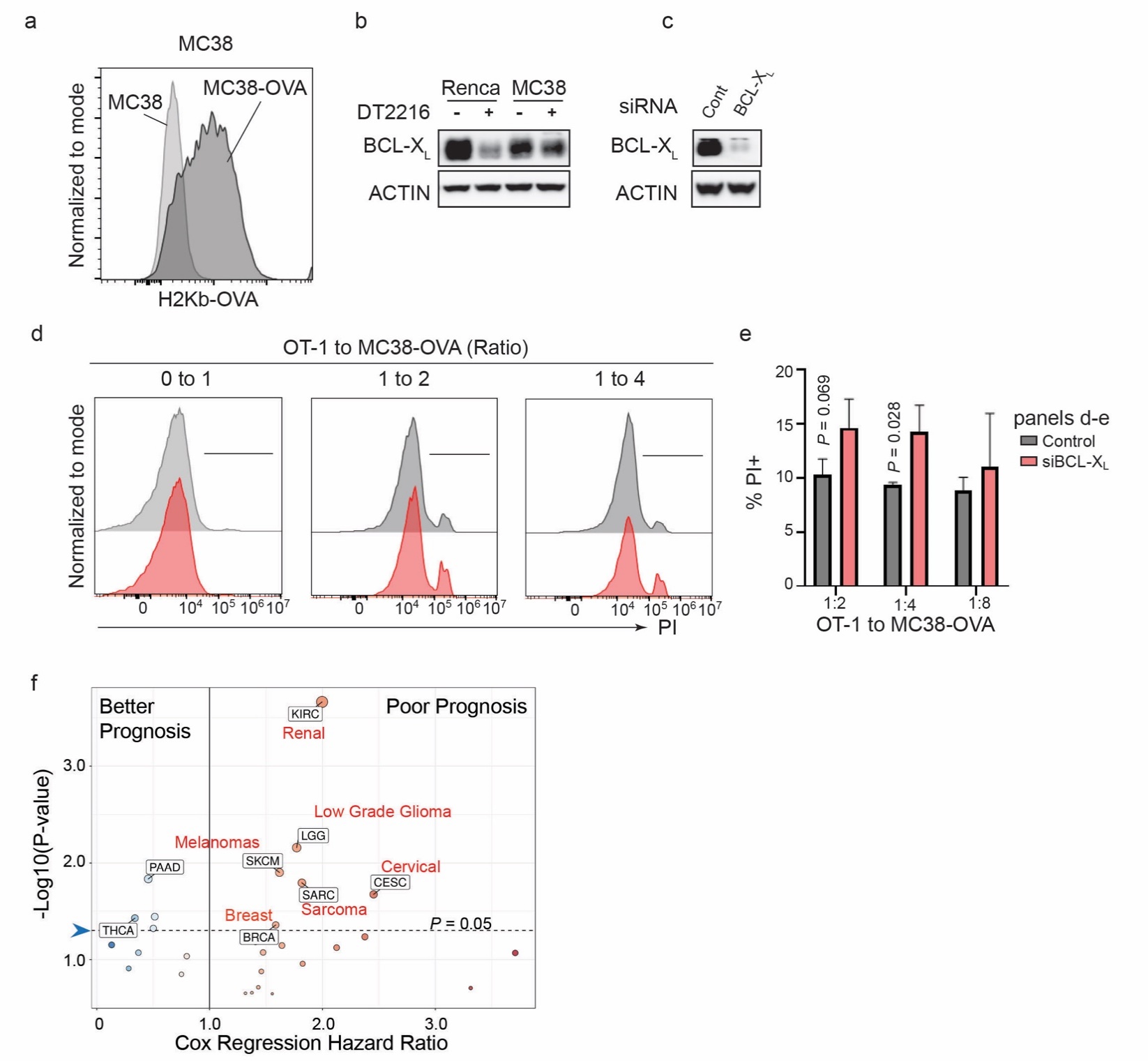


**Supplementary Figure 11. BCL-X_L_ protein expression is correlated with poor prognosis in human cancers.** Analysis of correlation between BCL-X_L_ protein expression and hazard ratios (HR) in the TCGA protein datasets. Logrank test was used. -log10 (*P*-values) are shown on the Y-axis and Cox regression HR are shown on the X-Axis. KIRC: kidney renal clear cell carcinoma (Renal); LGG: low-grade glioma; CESC: cervical squamous cell carcinoma and endocervical adenocarcinoma; SKCM: skin cutaneous melanoma; SARC: sarcoma; BRCA: breast invasive carcinoma; PAAD: pancreatic adenocarcinoma; THCA: thyroid carcinoma.


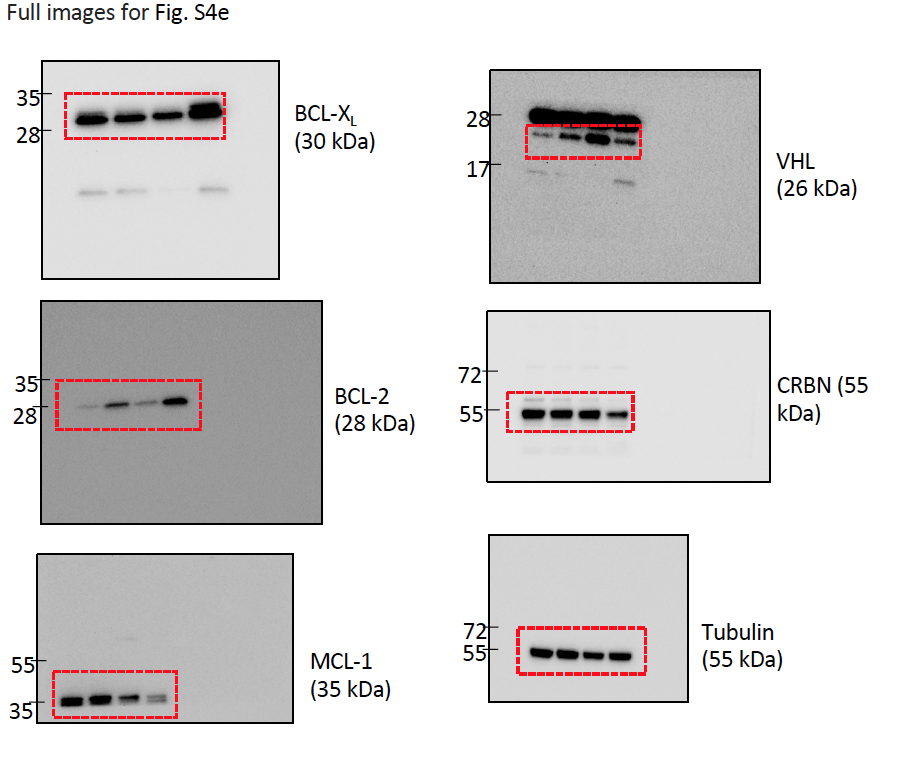


**Supplementary Figure 12. Full images for Supplementary Figure 4e.**


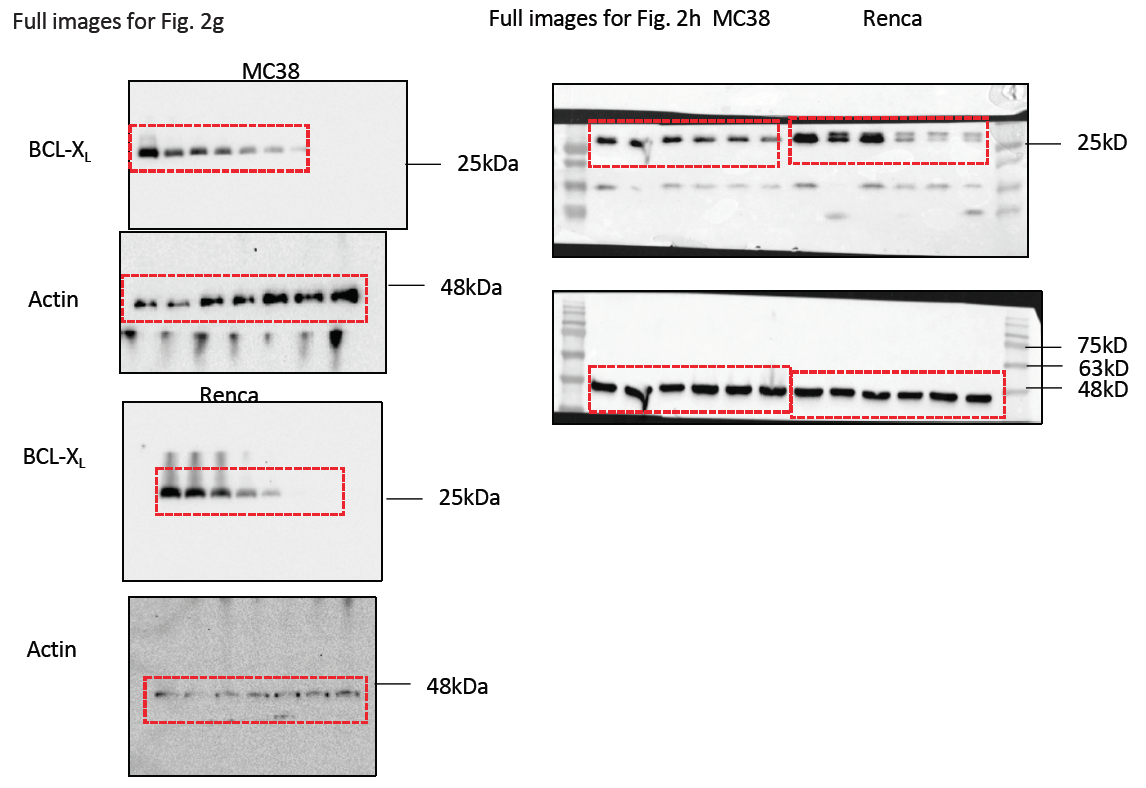


**Supplementary Figure 13. Full images for Fig.2g and Fig.2h.**


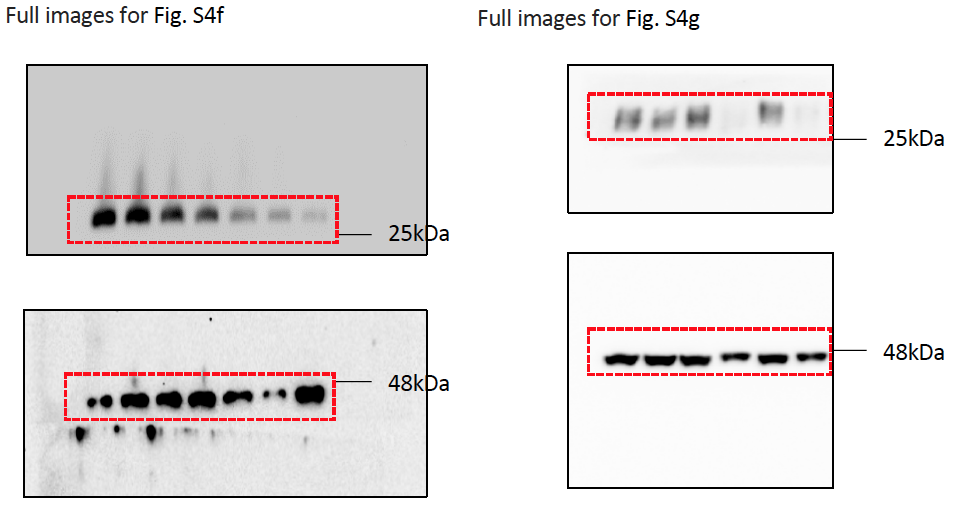


**Supplementary Figure 14. Full images for Supplementary Figure 4f-g.**


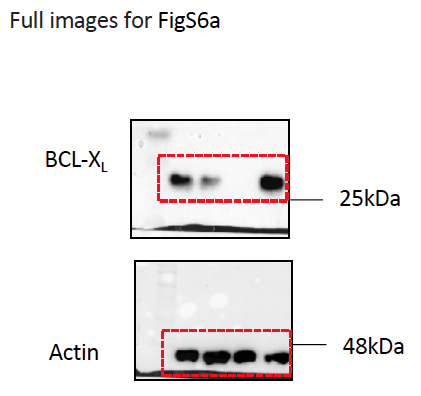


**Supplementary Figure 15. Full images for Supplementary Figure 6a.**
