# Supplementary material for: Proteolysis-targeting chimera against BCL-XL destroys tumor-infiltrating regulatory T cells
Source: Nat Commun. 2021 Feb 24;12:1281. doi: 10.1038/s41467-021-21573-x (PMC7904819; doi:10.1038/s41467-021-21573-x)
Supplement: Supplementary file 3 — Reporting Summary [file 41467_2021_21573_MOESM3_ESM.pdf]

nature research

Corresponding author(s): Weizhou Zhang
Daohong Zhou

Last updated by author(s): Dec 31, 2020

Reporting Summary

Nature Research wishes to improve the reproducibility of the work that we publish. This form provides structure for consistency and transparency in reporting. For further information on Nature Research policies, see Authors & Referees and the Editorial Policy Checklist .

Statistics

For all statistical analyses, confirm that the following items are present in the figure legend, table legend, main text, or Methods section.

n/a	Confirmed	
☐	☒	The exact sample size (*n*) for each experimental group/condition, given as a discrete number and unit of measurement
☐	☒	A statement on whether measurements were taken from distinct samples or whether the same sample was measured repeatedly
☐	☒	The statistical test(s) used AND whether they are one- or two-sided<br>*Only common tests should be described solely by name; describe more complex techniques in the Methods section.*
☒	☐	A description of all covariates tested
☐	☒	A description of any assumptions or corrections, such as tests of normality and adjustment for multiple comparisons
☐	☒	A full description of the statistical parameters including central tendency (e.g. means) or other basic estimates (e.g. regression coefficient) AND variation (e.g. standard deviation) or associated estimates of uncertainty (e.g. confidence intervals)
☐	☒	For null hypothesis testing, the test statistic (e.g. *F*, *t*, *r*) with confidence intervals, effect sizes, degrees of freedom and *P* value noted<br>*Give P values as exact values whenever suitable.*
☒	☐	For Bayesian analysis, information on the choice of priors and Markov chain Monte Carlo settings
☒	☐	For hierarchical and complex designs, identification of the appropriate level for tests and full reporting of outcomes
☒	☐	Estimates of effect sizes (e.g. Cohen's *d*, Pearson's *r*), indicating how they were calculated

Our web collection on statistics for biologists contains articles on many of the points above.

Software and code

Policy information about availability of computer code

Data collection	All codes for single cell analysis were deposited into GibHub by Dr. Borcherding. Link - https://github.com/ncborcherding/BCLXL
Data analysis	Data analysis was done by the Dr. Borcherding who developed all the codes and statistical analysis. Software used: FlowJo_v10.6.1; GraphPad Prism 8.3.0; sleuth R package (v0.30.0); monocle 2 R Package; SingleR R package V0.2.0; CellRanger v2.2 pipeline were used for all analysis.

For manuscripts utilizing custom algorithms or software that are central to the research but not yet described in published literature, software must be made available to editors/reviewers. We strongly encourage code deposition in a community repository (e.g. GitHub). See the Nature Research guidelines for submitting code & software for further information.

Data

Policy information about availability of data

All manuscripts must include a data availability statement. This statement should provide the following information, where applicable:

- Accession codes, unique identifiers, or web links for publicly available datasets
- A list of figures that have associated raw data
- A description of any restrictions on data availability

All original data and mouse models are available upon request. We included new analysis from publicly available datasets including: GSE8922520 and GSE9863818. The single-cell RNA sequencing result for renal cancer is deposited as GSE121638 and the TI-Tregs from MC38 tumor model is deposited as GSE150420.
All modified codes related to SCRC analysis are deposited at https://github.com/ncborcherding/BCLXL.

Field-specific reporting

Please select the one below that is the best fit for your research. If you are not sure, read the appropriate sections before making your selection.

☒ Life sciences ☐ Behavioural & social sciences ☐ Ecological, evolutionary & environmental sciences

For a reference copy of the document with all sections, see nature.com/documents/nr-reporting-summary-flat.pdf

Life sciences study design

All studies must disclose on these points even when the disclosure is negative.

Sample size	Sample size is used depending on individual experiments, based on variation, statistical power and the nature of the experiments. For example tumor studies with expectation of 1 fold variation and power 0.8, we estimate 6-7 mice per group. For other experiments, we don't have estimate of group variations and record data as we performed individual experiments. Statistics were done using GraphPad Prism 8.3.0. For tumor studies, a two-way ANOVA was used; for other comparisons, unpaired T test were used; for single cell RNAseq data or RNAseq data, we used Bonferroni adjusted P values.
Data exclusions	All data is included, unless flow data when no cells were detected due to technical error.
Replication	All experiments were repeated at least once with the number of mice or data points per group and successful in supporting the results
Randomization	All animal experiments were randomized. For mice receiving different treatment, we routinely put all mice into the same container and separate them out randomly into groups for different treatment. For other experiments related to flow cytometry, the randomization was at the stage of mice separation as above. For human samples, randomization didn't apply and we used whatever samples we could get from collection.
Blinding	All tumor studies are double blinded. The drug treatment is from one postdoc/technician and the tumor measurement is from a different postdoc/technician. To avoid technical error, one person sticks to one particularly data acquisition. For example, the same person will continue to monitor tumor growth for the same cohort of experiments. For other experiments, blinding is impossible and there is no step that can introduce personal bias.

Reporting for specific materials, systems and methods

We require information from authors about some types of materials, experimental systems and methods used in many studies. Here, indicate whether each material, system or method listed is relevant to your study. If you are not sure if a list item applies to your research, read the appropriate section before selecting a response.

Materials & experimental systems

n/a	Involved in the study	
☐	☒	Antibodies
☐	☒	Eukaryotic cell lines
☒	☐	Palaeontology
☐	☒	Animals and other organisms
☐	☒	Human research participants
☒	☐	Clinical data

Methods

n/a	Involved in the study	
☒	☐	ChIP-seq
☐	☒	Flow cytometry
☒	☐	MRI-based neuroimaging

Antibodies

Antibodies used	We used many antibodies, all from well validated companies, such as WB antibodies are most from Cell Signaling/Abcam; and flow antibodies are mostly from Biolegend, unless otherwise noted: Anti-mouse CD62L-BV785 (clone MEL-14), anti-mouse MHCII I-A/I-E-BB515 (Clone 2G9, BD Biosciences), anti-mouse CD11B-PEdazzle (clone M1/70), anti-mouse CD45-AF532 (clone 30F.11), anti-mouse CD3-APC (clone 17A2), anti-mouse CD8-BV510 (clone 53-6.7), anti-mouse CD4-BV605 (clone GK1.5), anti-mouse NK1.1-AF700 (clone PK136), anti-mouse CD69-SB436 (clone H1.2F3, eBioscience), anti-mouse CD279 (clone-PerCP-EF710, eBioscience Inc), anti-mouse CD366-PacBlue (clone B8.2c12), anti-mouse CD11C-PE-Cy7 (clone N418), anti-mouse Ly6G-FITC (clone IA8), anti-mouse Ly6C-BV711 (clone HK1.4) anti-mouse F4/80-BV650 (clone BM8), anti-mouse CD80-BV480 (clone 16-10A1, BD Biosciences), anti-mouse CD25-PE-Cy5 (clone PC61), FVD-eFluor-780 (eBioscience) and mouse FcR blocker (anti-mouse CD16/CD32, clone 2.4G2, BD Biosciences), anti-mouse FOXP3-APC (clone FJK-16S, eBioscience), anti-mouse Granzyme B-Pacific Blue (clone GB11), anti-mouse Perforin-PE (clone S16009B), anti-mouse Ki-67-PerCP-Cy5.5 (clone 16A8), anti-mouse/human BCL-XL-PE (clone S486; Cell Signaling Technologies, Danvers, MA). Human cells were stained with a combination of the following antibodies: anti-human CD45-BV510 (clone H130), anti-human CD3-AF700 (clone HIT5a), anti-human CD4-BV421 (clone OKT4), anti-human CD8-BV711 (clone RPA-T8), anti-human CD127-BV605 (clone A019DS), anti-human CD25-PE-Cy7 (clone MA251) plus FVD-eFluor-780 (eBioscience) and human FcR blocking Reagent (StemCell Technologies), anti-human FOXP3-FITC (clone 206D), anti-mouse/human BCL-XL-PE (clone S486, Cell Signaling Technologies). All standard staining procedure of 1:100 or 1:200 dilution.

Validation

Most of these antibodies are used hundreds of times in literature and validated from a lot publications. Company validates each batch before selling. The lab routinely validates new antibodies using various approaches.

FC (mouse) clone Application notes Validation Reference

CD45 30F.11 reacts with all isoforms and CD45.1 and CD45.2 alloantigens of CD45 Flow Cytometry - quality tested Podd B et al. 2006 J Immunol 176:6532

CD3 17A2 Flow Cytometry - quality tested Xiao S, et al. 2007 J Exp Med. 204:1691

CD4 GK1.5 Flow Cytometry - quality tested Dialyns DP, et al. Immunol. 131:2445

CD25 PC61 Flow Cytometry - quality tested Galitovskiy V, et al. 2011. J Immunol. 187.2677

GITR DTA1 Flow Cytometry - quality tested MaruYama T, et al. 2015. J Leukoc Biol. 98:385-393

CD62L MEL-14 Flow Cytometry - quality tested Leddon S, Sant A. 2012 PloS One. 7e46952

MHCII I-A/I-E Clone 2G9 Also reacts with I-Eb and I-Ek and with cells from mice of the H-2p and H-2q haplotypes non Routinely tested by flow cytometry Becker D, et al. 1999 J Invest Dermatol. 99(5):545-549

cd11b M1/70 mouse, human. Cross reacts Chimpanzee, Babboon, Cynomolgus, Rhesus, Rabbit Flow Cytometry - quality tested Prislovsky A, Strom T. 2013 Exp Hematol. 41:789

cd8 53-6.7 Flow Cytometry - quality tested Lee C, et al. 2009. Clin Cancer Res. 2.538888889

NK1.1 PK136 Flow Cytometry - quality tested Kroemer A, et al. 2008. J Immunol. 180:7818

Cd69 H1.2F3 augment T cell activatgion. In Vitro T cell and NK cell activation Flow Cytometry - quality tested Mitri D, et al. 2011. J Immunol. 187:2093

CD279 29F.1A12 Flow Cytometry - quality tested Smith T, et al. 2014. J Immunol. 11:535

CD366 B8.2c12 only recognizes the Balb/c Tim-3 allele Flow Cytometry - quality tested Mizukami S, et al. 2015. Proc Natl Acad Sci USA. 112:1809

CD11C N418 Flow Cytometry - quality tested Roland C, et al. 2009 Mol Cancer Ther. 1.55625

Ly6G 1A8 Only recognized Ly6G, not Ly6C Flow Cytometry - quality tested Georgoudaki A, et al. 2016. Cell Rep. 15:2000-2011

Ly6G HK1.4 Flow Cytometry - quality tested Alexander J, et al. 2012. Kidney Int. 82:961

F4/80 BM8 Flow Cytometry - quality tested Bian Z, et al. 2012. J Immunol. 188:844

CD80 16-10A1 Flow Cytometry - quality tested Philipsen L, et al. 2013. Mol Cell Proteomics. 12:2551

CD25 PC61 Flow Cytometry - quality tested Galitovskiy V, et al. 2011. J Immunol. 187.2677

FOXP3 FJK-16S reacts with mouse, rat, dog, procine, bovine and cat FOXP3 This antibody was verified by Relative expression to ensure that the antibody binds to the antigen stated, Tested by intracellular flow cytometry Yang WC, et al. 2017. Front Immunol. 8:1508

GZMB GB11 Reactivity: human, mouse. Cross-reactivity: rat each lot is quality contol tested by intracellular immunofluorescent staining with flow cytometric analysis Srivastava R, et al. 2015. J Immunol. 194:2232

Perforin S16009B reacts with both C57Bl/6 and Balb/c strains each lot is quality contol tested by intracellular immunofluorescent staining with flow cytometric analysis

KI-67 16A8 each lot is quality control tested by Ki-67 protocol Davidson S, et al.2020. Cell Rep. 31:107628

CD44 IM7 Reactivity: mouse, human Flow Cytometry - quality tested McClellan S, et al. 2015. Methods. 82:47-54

CD39 Duha59 Flow Cytometry - quality tested

CD73 Rty/11.8 Flow Cytometry - quality tested Li L, et al. 20147. FASEB J. 31:1067

BCL-Xl 54H6 Reactis with human and mouse. Same clone withouth conjugation was used for western blots met all of the quality control standards defined by Cell Signaling Technologiy, Inc Ludwig LM, et al. 2018 Methods Mol Biol. 1877:77-91 Anukriti S, et al. 2020. Neoplasia. 22(10): 497-510

FC (Human) clone Application notes Validation Reference

CD45 HI30 Flow Cytometry - quality tested Bashour K, et al. 2014. Proc Natl Acad Sci USA. 111:2241

CD3 HIT3a Flow Cytometry - quality tested Lopez-Verges S, et al. 2010. Blood, 116:3865

CD4 OKT4 Flow Cytometry - quality tested Alman K 2006. J immunol. 177:1721

CD8a RPA-T8 Flow Cytometry - quality tested Dettling S, et al. 2018. Clin Cancer Res. 24(12)

CD127 A019D5 Flow Cytometry - quality tested Anginari S, et al. 2020. Cell Metab. 31:391

CD25 M-A251 Recognizes Epitope B of CD25 Flow Cytometry - quality tested Jin J, et al. 2014. PloS One. 9:104753

FOXP3 206D each lot is quality contol tested by intracellular flow cytometry using the True-Nuclear Transcription factor staining protocol Liu s, et al. 2006. J Exp Med. 203:1701

CD45RA H10D Flow Cytometry - quality tested Marquadt N, et al. 2015. J Immunol. 194:2467

CCR7 G043H7 Flow Cytometry - quality tested Jurchott K, et al. 2016. PloS One. 11:0150812

Western Blot Clone Application notes Validation Reference

β-actin 8H10D10 Reactivity: Mouse, human, rat met all of the quality control standards defined by Cell Signaling Technologiy, Inc Zhao F, et al. 2020. J Exp Med. 20(5):68

MCL-1 D35A5 Reactivity: Mouse, human met all of the quality control standards defined by Cell Signaling Technologiy, Inc Nguyen TTT, et al. 2020. Cells. 9(7):1661

BCL-2 50E3 Reactivity: Mouse, human, rat met all of the quality control standards defined by Cell Signaling Technologiy, Inc (product discontinued) Yang W, et al. 2020. Cancers. 12(5):1329

VHL polyclonal Reactivity: Mouse, human, rat Zhang WC, et al. 2019. Nat Metab. 1(4):460-474

CRBN polyclonal Reactivity: Mouse, human, rat Validated for western blot and immunohistochemistry

β-Tubulin polyclonal Reactivity: Mouse, human, rat met all of the quality control standards defined by Cell Signaling Technologiy, Inc Chen Y,et al. 2020. Mol Med Rep. 22(3):2342-2350

Eukaryotic cell lines

Policy information about cell lines

Cell line source(s)	All parental cell lines including MC38, 4T1, and RENCA are from ATCC. PY8119 is from Dr. Lesley before she provided to ATCC.
Authentication	All cells are frozen a large batch from ATCC or from collaborators. They haven't been passaged a lot. We normally use morphological feature for distinct cell lines, ability to form tumors and histology of these tumors. We only use cell lines within 6 passages from ATCC if possible. All cell lines were not authenticated using STR profiling in the laboratory.
Mycoplasma contamination	All parental cell lines were tested negative for mycoplasma and other known mouse pathogens using Mouse Essential CLEAR Panel from Charles River.
Commonly misidentified lines (See ICLAC register)	No commonly misidentified cell lines were used in the study.

Animals and other organisms

Policy information about studies involving animals; ARRIVE guidelines recommended for reporting animal research

Laboratory animals	Mouse: Strain: C57BL/6N from Charles River, 6-8 weeks of age, males for MC38 and females for Py8119 tumors; BalB/C, Charles Rivers, 6-8 weeks of age, males for RENCA.
Wild animals	No wild animals were used in the study.
Field-collected samples	No field collected samples were used in the study.
Ethics oversight	University of Florida IACUC

Note that full information on the approval of the study protocol must also be provided in the manuscript.

Human research participants

Policy information about studies involving human research participants

Population characteristics	The patients were males with an age range of 67 to 74 years old. Tumor grades were histologically determined by a pathologist. Primary tumor stages for Patient 1 and Patient 2 were reported as pT1b without extension, while Patient 3 was reported as pT3a with renal vein invasion. Paired blood and primary ccRCC along with matched normal kidney parenchyma samples were obtained from the University of Iowa Tissue Procurement Core and GUMER repository through the Holden Comprehensive Cancer Center from de-identified three subjects previously provided written consent approved by the University of Iowa Institutional Review Board (IRB) under the IRB number 201304826 and conducted under the Declaration of Helsinki Principles.
Recruitment	Patients recruitment was through the University of Iowa Tissue Procurement Core and GUMER repository from the Holden Comprehensive Cancer Center, de-identified with written consents.
Ethics oversight	University of Iowa Institutional Review Board (IRB) under the IRB number 201304826. Other human tissues were collected under IRB protocol: 201901677 that was approved by the University of Florida Institutional Review Board as non-human subject protocol

Note that full information on the approval of the study protocol must also be provided in the manuscript.

Flow Cytometry

Plots

Confirm that:

- [x] The axis labels state the marker and fluorochrome used (e.g. CD4-FITC).
- [x] The axis scales are clearly visible. Include numbers along axes only for bottom left plot of group (a 'group' is an analysis of identical markers).
- [x] All plots are contour plots with outliers or pseudocolor plots.
- [x] A numerical value for number of cells or percentage (with statistics) is provided.

Methodology

Sample preparation	Tumors, spleen, lymph nodes, bloods were routinely used. Tumors were excised and approximately 200 mg of tumor tissue were enzymatically and mechanically digested using the mouse Tumor Dissociation Kit (Miltenyi Biotec) to obtain a single cell suspension. Human tumor samples and sections were enzymatically and mechanically digested using the human Tumor Dissociation Kit (Miltenyi Biotec) to obtain single cell suspension. Red blood cells were lysed using ACK lysis buffer and mononuclear cells were isolated by density gradient using SepMate Tubes (StemCell Technologies) and Lymphoprep density gradient media (StemCell Technologies). Cells were then washed and incubated with combinations of antibodies for staining and

intracellular staining.

Instrument	LSRII and Aurora Cytek
Software	Flowjo
Cell population abundance	Please note the projects did not use post-sort cells for further experiments. We only used analytical flow cytometry to analyze cell populations within different tissues.
Gating strategy	All gating started with a SSC-H and FSC-H gating for lymphocytes, directed by splenocytes or blood lymphocyte populations. We increased gating size to include granulocytes and monocytes for myeloid gating. For single cell gating, we did two steps including SSC-H versus SSC-A; and FSC-H versus FSC-A. Next step is to gate on only live cells using fixable viability dye eFluor780 (or other FVD with different fluorophores) from Ebiosciences. We gated on CD45+ cells and then CD3+ T cells, following specific gating on CD4, and CD8 T cells. We used CD4+FoxP3+ to define Tregs in most of the studies.

☒ Tick this box to confirm that a figure exemplifying the gating strategy is provided in the Supplementary Information.
